# RETRACTION: The Relationship Between Vitamin D Deficiency and Diabetic Foot Ulcer: A Meta‐Analysis

**DOI:** 10.1111/iwj.70202

**Published:** 2025-02-02

## Abstract

**RETRACTION:** LiX. , KouS. , ChenG. , ZhaoB. , XueJ. , DingR. , ZhaoX. , YeM. , YangY. , YueR. , and LiF. , “The Relationship Between Vitamin D Deficiency and Diabetic Foot Ulcer: A Meta‐Analysis,” International Wound Journal 20, no. 8 (2023): 3015–3022, 10.1111/iwj.14177.37194326 PMC10502267

The above article, published online on 16 May 2023, in Wiley Online Library (http://onlinelibrary.wiley.com/), has been retracted by agreement between the journal Editor in Chief, Professor Keith Harding; and John Wiley & Sons Ltd. Following an investigation by the publisher, all parties have concluded that this article was accepted solely on the basis of a compromised peer review process. The editors have therefore decided to retract the article. The authors disagree with the retraction.

**RETRACTION:** X. Li , S. Kou , G. Chen , B. Zhao , J. Xue , R. Ding , X. Zhao , M. Ye , Y. Yang , R. Yue , and F. Li , “The Relationship Between Vitamin D Deficiency and Diabetic Foot Ulcer: A Meta‐Analysis,” International Wound Journal 20, no. 8 (2023): 3015–3022, 10.1111/iwj.14177.37194326 PMC10502267

The above article, published online on 16 May 2023, in Wiley Online Library (http://onlinelibrary.wiley.com/), has been retracted by agreement between the journal Editor in Chief, Professor Keith Harding; and John Wiley & Sons Ltd. Following an investigation by the publisher, all parties have concluded that this article was accepted solely on the basis of a compromised peer review process. The editors have therefore decided to retract the article. The authors disagree with the retraction.

